# Acacia Polyphenol Ameliorates Atopic Dermatitis in Trimellitic Anhydride-Induced Model Mice via Changes in the Gut Microbiota

**DOI:** 10.3390/foods9060773

**Published:** 2020-06-11

**Authors:** Nobutomo Ikarashi, Natsumi Fujitate, Takumi Togashi, Naoya Takayama, Natsuko Fukuda, Risako Kon, Hiroyasu Sakai, Junzo Kamei, Kiyoshi Sugiyama

**Affiliations:** 1 Department of Biomolecular Pharmacology, Hoshi University, 2-4-41 Ebara, Shinagawa-ku, Tokyo 142-8501, Japan; s141223@hoshi.ac.jp (N.F.); s151171@hoshi.ac.jp (T.T.); s151150@hoshi.ac.jp (N.T.); s151206@hoshi.ac.jp (N.F.); r-kon@hoshi.ac.jp (R.K.); sakai@hoshi.ac.jp (H.S.); kamei@hoshi.ac.jp (J.K.); 2 Department of Functional Molecular Kinetics, Hoshi University, 2-4-41 Ebara, Shinagawa-ku, Tokyo 142-8501, Japan

**Keywords:** acacia polyphenol, atopic dermatitis, gut microbiota, functional food

## Abstract

We have previously shown that acacia polyphenol (AP), which was extracted from the bark of *Acacia mearnsii* De Wild, exerts antiobesity, antidiabetic, and antihypertensive effects. In this study, we examined the effect of AP on atopic dermatitis. Trimellitic anhydride (TMA) was applied to the ears of mice to create model mice with atopic dermatitis. The frequency of scratching behavior in the TMA-treated group was significantly higher than that in the control group, and the expression levels of inflammatory markers (tumor necrosis factor-α, interleukin-6, inducible nitric oxide synthase, and cyclooxygenase-2) in the skin also increased. In contrast, both the frequency of scratching behavior and the expression levels of skin inflammatory markers in the AP-treated group were significantly lower than those in the TMA-treated group. The abundances of beneficial bacteria, such as *Bifidobacterium* spp. and *Lactobacillus* spp., increased in the AP-treated group compared with the TMA-treated group. Furthermore, the abundances of *Bacteroides fragilis* and *Clostridium coccoides* in the gut, which are known for anti-inflammatory properties, increased significantly with AP administration. The present results revealed that AP inhibits TMA-induced atopic dermatitis-like symptoms. In addition, the results also suggested that this effect may be associated with the mechanism of gut microbiota improvement.

## 1. Introduction

Atopic dermatitis causes eczema, accompanied by itchiness on the body, with repeated aggravation and remission. As the quality of life of patients with atopic dermatitis is markedly decreased due to itchiness, the development of a suitable treatment is very important. However, there are still many unclear points about the pathogenic mechanism of atopic dermatitis. Currently, in the treatment of atopic dermatitis, topical steroids and immunosuppressants are used as symptomatic treatments, but it is not a fundamental treatment.

In recent years, it has become clear that gut microbiota is involved in the development of atopic dermatitis [1]. For example, it has been reported that (1) patients with atopic dermatitis had more intestinal *Clostridium* spp. and fewer intestinal *Bifidobacterium* spp. in their feces than non-atopic dermatitis subjects [2] and that (2) the administration of probiotics improved atopic dermatitis [3,4,5,6]. Thus, drugs or foods that correct the gut microbiota may be useful for atopic dermatitis. To date, we have clarified that polyphenols, which have a poor absorption rate in the digestive tract, change the flora in the large intestine, resulting in substantial alteration of hepatic functional molecules [7,8]. Thus, it was considered that polyphenols may improve atopic dermatitis through the change of gut microbiota, as well as probiotics.

The aqueous extract from the bark of *Acacia mearnsii* De Wild. (acacia polyphenol (AP)) is high in polyphenols, and we have previously shown that AP exerts antiobesity, antidiabetic, and antihypertensive effects [9,10,11]. In addition, we have demonstrated that AP ameliorates atopic dermatitis-like symptoms in mice fed a magnesium-free special diet [12]. However, little is known about the mechanism by which AP improves atopic dermatitis, and, in particular, the effect on gut microbiota is not known at all. Therefore, based on the above findings, we hypothesized that AP changed gut microbiota and improved atopic dermatitis and verified the hypothesis. Briefly, AP was administered to trimellitic anhydride (TMA)-induced atopic dermatitis model mice [13], after which their scratching behavior and skin inflammatory reactions were observed. In addition, the abundances of gut microbes in the feces were analyzed.

## 2. Materials and Methods

### 2.1. The Preparation of AP

AP (lot: DF5) was donated by Acacia-No-Ki Co., Ltd. (Hiroshima, Japan) and prepared according to Cutting’s methods [14]. Briefly, the bark of *A. mearnsii* De Wild. from South Africa was crushed, extracted with hot water (100 °C), and dried with a spray drier. The HPLC chart of AP used in this study is shown in Figure S1. Details of AP, such as the ingredients, were given in previous reports [15,16,17].

### 2.2. Animals

Female BALB/c mice (5 weeks old, Japan SLC, Inc., Shizuoka, Japan) were used in this study. The mice were housed at 55 ± 5% humidity and room temperature (24 ± 1 °C). The study was conducted with approval (approval no. 30–121) and in accordance with the Hoshi University Guiding Principles.

### 2.3. TMA-Induced Atopic Dermatitis

**Figure 1:**
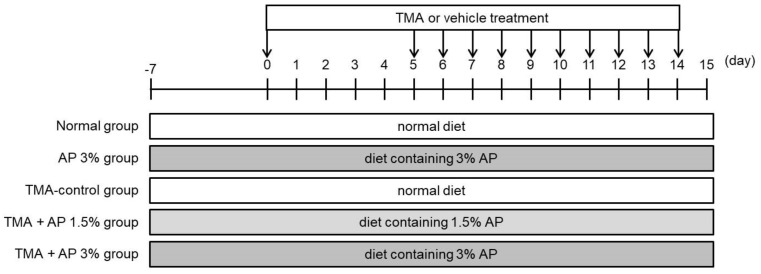
Experimental protocol.

The experimental protocol is illustrated in Figure 1 [13]. Briefly, mice were sensitized with TMA (Fujifilm Wako Pure Chemical Corporation, Osaka, Japan) by topical application on abdominal skin on day 0, received TMA on both ears on day 5, and were repeatedly challenged with TMA on days 6–14. The vehicle groups were treated with a mixture of acetone and isopropylmyristate (4:1, *v*/*v*).

### 2.4. Treatments

The mice were divided into the following groups (*n* = 8, 4 mice/cage, Figure 1): (1) normal group, (2) AP 3% group, (3) TMA-control group, (4) TMA-AP 1.5% group, and (5) TMA-AP 3% group. With the exception of the mice in the normal and AP 3% groups, all mice were treated with TMA to induce atopic dermatitis (AD)-like skin lesions. Each mouse was fed a normal diet (MF, Oriental Yeast, Tokyo, Japan) or a normal diet containing 1.5% or 3% AP ad libitum for 22 days. On the 15th day (Figure 1), the ear skin and feces in the colon were removed and put in tubes. The samples were flash-frozen in liquid nitrogen and stored at −80 °C until analysis.

### 2.5. Scratching Behavior

Scratching behavior was observed on the 14th day (Figure 1). To measure scratching behavior, mice were individually housed in a cage divided into compartments and acclimatized for 60 min. The scratching behavior was recorded using a digital video camera for 30 min, with no direct human observation. The number of scratching episodes was counted from the recordings.

### 2.6. Hematoxylin-Eosin (HE) Staining

After mouse skin was fixed and embedded in paraffin, the sample was cut into 5-μm sections and mounted on slides. The slides were stained with hematoxylin and eosin. We observed the epidermal thickness, presence of ulcer, and infiltration of inflammatory cells into the dermis.

### 2.7. Real-Time RT-PCR

Total RNA from mouse skin was isolated by homogenization in TRI reagent (Sigma-Aldrich Corp., St. Louis, MO, USA). cDNA was synthesized by a high-capacity cDNA synthesis kit (Applied Biosystems, Foster City, CA, USA). Target gene expression was analyzed by real-time RT-PCR (CFX Connect Real-Time System, Bio-Rad Laboratories, Hercules, CA, USA). The primer sequences for real-time PCR are shown in Table 1. The mRNA expressions were normalized to the expression of the housekeeping gene 18S rRNA.

### 2.8. Quantification of Bacteria from Fecal Samples

DNA from the feces was extracted by the QIAamp DNA Stool Mini Kit (Qiagen, Inc., Valencia, CA, USA). Target gene expression was analyzed by real-time RT-PCR. The primer sequences for the gut microbiota are shown in Table 2. The DNA expression levels were normalized to the expression of 16S rRNA.

### 2.9. Statistical Analysis

The results are presented as the mean ± standard deviation. The Tukey’s test was used to compare data among groups. Values of *p* < 0.05 were considered to indicate significance.

## 3. Results

### 3.1. Scratching Behavior

**Figure 2:**
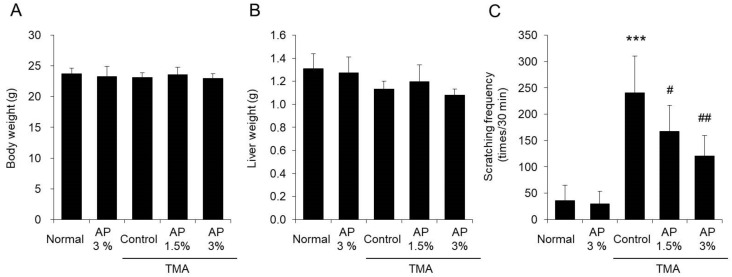
Scratching behavior. Mice with atopic dermatitis induced by repeated application of TMA were given a diet with AP. The body weight (**A**), liver weight (**B**), and scratching behavior (**C**) were measured (mean ± SD, N = 8, *** *p* < 0.001 vs. normal group, # *p* < 0.05 vs. TMA-control group, ## *p* < 0.01 vs. TMA-control group).

We examined the effect of AP on scratching behavior (Figure 2). When AP was administered to mice, no changes occurred in either the body or liver weight (Figure 2A,B). The frequency of scratching behavior in the TMA-control group was approximately 250 times per 30 min, which was approximately 10 times higher than that in the normal group. In contrast, the frequency of scratching behavior in the TMA+AP 1.5% group was approximately 180 times per 30 min, while that in the TMA+AP 3% group was approximately 130 times per 30 min, showing that AP exhibited a significant dose-dependent reduction in scratching behavior (Figure 2C).

The above results demonstrate that AP inhibits the scratching behavior induced by TMA.

### 3.2. Skin Inflammation

**Figure 3:**
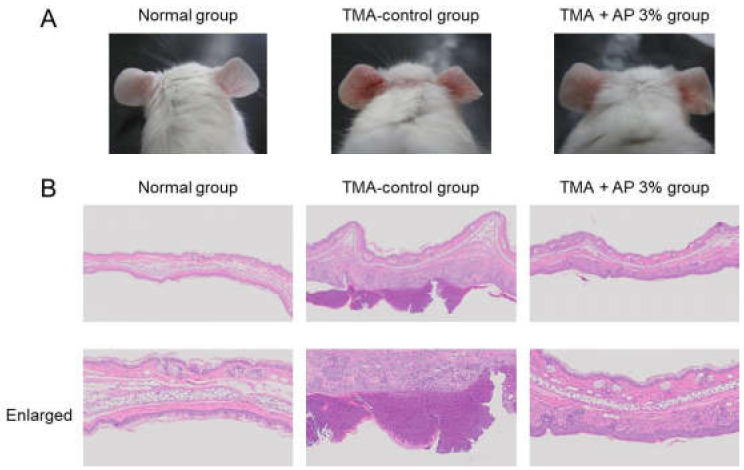
Skin inflammation. Mice with atopic dermatitis induced by repeated application of TMA were given a diet with AP. Clinical features of skin were observed (**A**). Skin tissue was assessed by HE staining (**B**).

**Figure 4:**
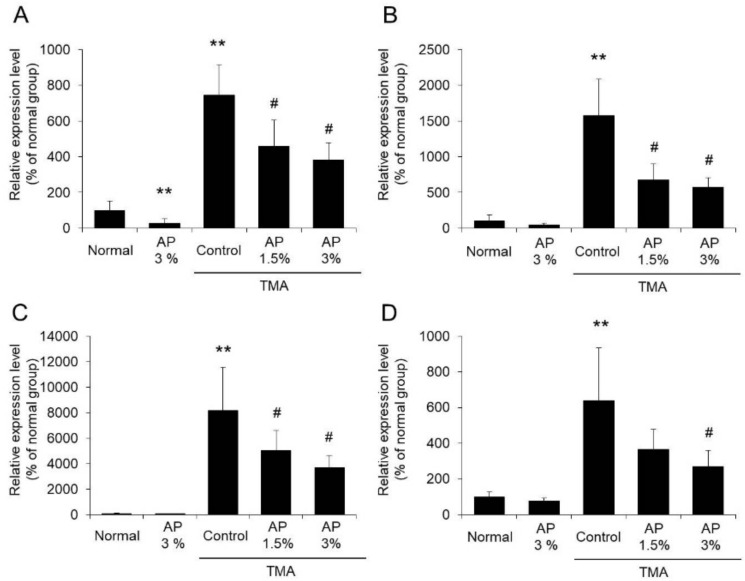
The mRNA expression levels of TNF-α (**A**), IL-6 (**B**), iNOS (**C**), and COX-2 (**D**). Mice with atopic dermatitis induced by repeated application of TMA were given a diet with AP. The skin was harvested, and the TNF-α (**A**), IL-6 (**B**), iNOS (**C**), and COX-2 (**D**) mRNA expression levels were measured using real-time RT-PCR (mean ± SD, N = 8, ** *p* < 0.01 vs. normal group, # *p* < 0.05 vs. TMA-control group).

At the onset of atopic dermatitis, skin inflammation occurs and the expression levels of inflammatory markers, such as TNF-α, IL-6, iNOS, and COX-2, increase [26,27]. We observed the skin condition and the effect of AP on the inflammatory reactions using these expression levels as indexes (Figure 3 and Figure 4).

Redness was noted in the ears of the TMA-control group mice compared with the normal group, and epidermal thickening, ulcers, and inflammatory cell infiltration into the dermis was observed. In contrast, improvement of these effects was noted in the TMA+AP 3% group compared with the TMA-control group (Figure 3).

The mRNA expression levels of TNF-α, IL-6, iNOS, and COX-2 in the ears of the TMA-control group increased markedly compared with the levels in the normal group. In contrast, the expression levels of TNF-α, IL-6, iNOS, and COX-2 in the ears of mice that were administered AP were significantly decreased compared with the levels in the TMA-control group (Figure 4).

Based on these findings, it is suggested that AP inhibits TMA-induced inflammatory reactions.

### 3.3. The Phyla Firmicutes, Bacteroidetes, Proteobacteria, and Actinobacteria

Recently, it has become clear that changes in the gut microbiota are associated with the onset and remission of atopic dermatitis [1,3,4,5,6]. Therefore, we investigated the mechanism by which AP inhibited atopic dermatitis, focusing on the intestinal flora.

**Figure 5:**
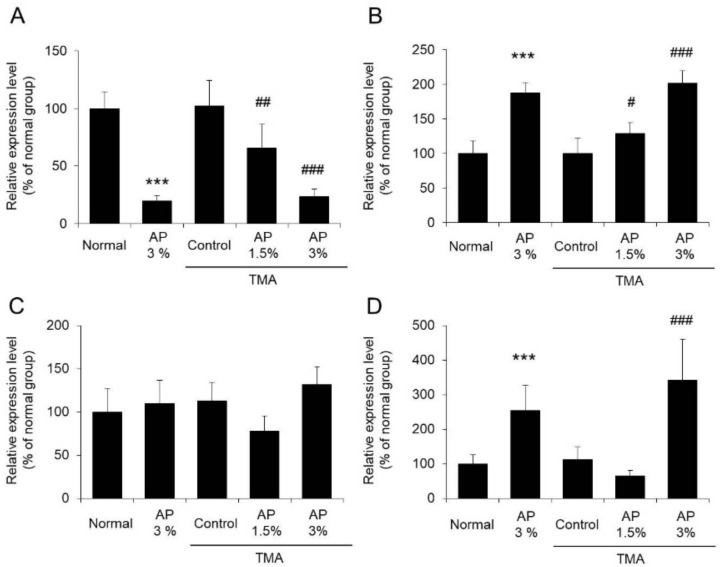
The phyla *Firmicutes* (**A**), *Bacteroidetes* (**B**)*, Proteobacteria* (**C**), and *Actinobacteria* (**D**). Mice with atopic dermatitis induced by repeated application of TMA were given a diet with AP. The feces were collected from the colon, and the abundances of the phyla *Firmicutes* (**A**), *Bacteroidetes* (**B**), *Proteobacteria* (**C**), and *Actinobacteria* (**D**) in the gut microbiota were measured using real-time PCR (mean ± SD, N = 8, *** *p* < 0.001 vs. normal group, # *p* < 0.05 vs. TMA-control group, ## *p* < 0.01 vs. TMA-control group, ### *p* < 0.001 vs. TMA-control group).

Ninety-nine percent of human intestinal bacteria belong to four phyla: *Firmicutes, Bacteroidetes, Proteobacteria*, and *Actinobacteria* [28,29]. The abundance of the gut microbiota (phyla *Firmicutes, Bacteroidetes, Proteobacteria*, and *Actinobacteria*) in the TMA-control group was approximately the same as that in the normal group. In contrast, administration of AP significantly decreased the abundance of the phylum *Firmicutes* in the gut microbiota and significantly increased that of the phyla *Bacteroidetes* and *Actinobacteria* (Figure 5).

Based on these findings, it is suggested that AP causes significant alterations in the gut microbiota.

### 3.4. Bifidobacterium spp., Lactobacillus spp., Bacteroides fragilis, and Clostridium coccoides

**Figure 6:**
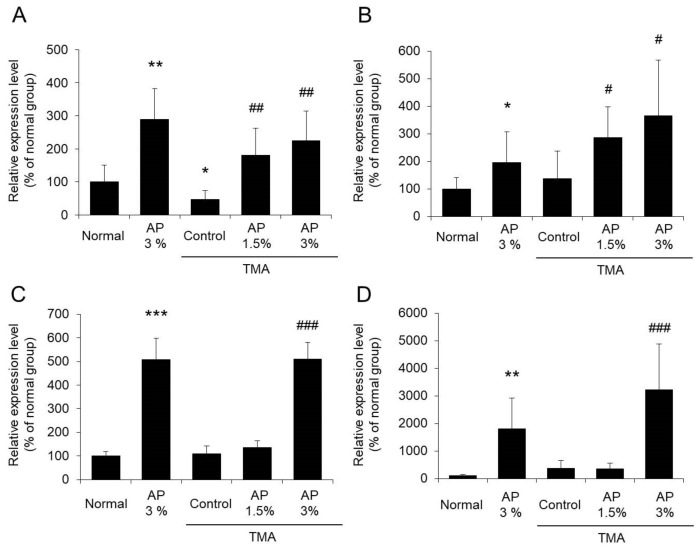
*Bifidobacterium* spp. (**A**), *Lactobacillus* spp. (**B**), *Bacteroides fragilis* (**C**), and *Clostridium coccoides* (**D**). Mice with atopic dermatitis induced by repeated application of TMA were given a diet with AP. The feces were collected from the colon, and the abundances of *Bifidobacterium* spp. (**A**), *Lactobacillus* spp. (**B**), *Bacteroides fragilis* (**C**), and *Clostridium coccoides* (**D**) in the gut microbiota were measured using real-time PCR (mean ± SD, N = 8, * *p* < 0.05 vs. normal group, ** *p* < 0.01 vs. normal group, *** *p* < 0.001 vs. normal group, # *p* < 0.05 vs. TMA-control group, ## *p* < 0.01 vs. TMA-control group, ### *p* < 0.001 vs. TMA-control group).

Gut microbes, such as *Bifidobacterium* spp. and *Lactobacillus* spp., are called beneficial bacteria and are considered to be effective in the prevention and treatment of many diseases [1,3,4,5,6]. Meanwhile, both *Bacteroides fragilis* and *Clostridium coccoides* are related to the onset and remission of inflammatory diseases [30,31,32]. The abundance of *Bifidobacterium* spp. in the gut microbiota decreased significantly in the TMA-control group compared with the normal group. In contrast, the abundance of *Bifidobacterium* spp. increased significantly in the AP-treated group in a dose-dependent manner compared with the TMA-control group (Figure 6A). No differences were noted in the abundance of *Lactobacillus* spp., *B. fragilis*, or *C. coccoides* in the gut microbiota between the normal group and the TMA-control group. The abundances of these bacteria increased significantly with the administration of AP (Figure 6B–D).

Based on the above findings, it is suggested that AP increases the abundances of *Bifidobacterium* spp., *Lactobacillus* spp., *B. fragilis*, and *C. coccoides*.

## 4. Discussion

AP is a powder extracted from the bark of *A. mearnsii* De Wild. by using hot water. It contains large amounts of flavonoids, such as fisetinidol and robinetinidol [15,16,17], and its antidiabetic, antiobesity, and antihypertensive effects have been confirmed [9,10,11]. In this study, we examined the effect of AP on atopic dermatitis and the underlying mechanism.

Model mice with atopic dermatitis exhibiting scratching behavior can be generated by regularly applying TMA to the ears of the mice. The mice used in this study also exhibited increased scratching behavior, as observed in previous reports [6]. When AP was administered to model mice with TMA-induced atopic dermatitis, inhibition of scratching behavior was observed (Figure 2). Although inflammatory reactions on the skin were noted in these model mice, AP significantly inhibited these reactions (Figure 3 and Figure 4). As it has been reported that the induction of skin inflammation increases the intensity of itchiness, there is a close relation between inflammatory reactions and scratching behavior [33]. Thus, these findings suggest that AP inhibits skin inflammatory reactions and restrains scratching behavior.

It is commonly known that polyphenols have a low rate of absorption. Thus, although we have not conducted a pharmacokinetic study of AP, it is considered that components containing AP are unlikely to affect the skin after being absorbed into the body. Meanwhile, we have confirmed that polyphenols change the flora in the large intestine, resulting in substantial alteration of hepatic functional molecules [7,8]. Therefore, we considered that there is a possibility that AP changes the gut microbiota and inhibits symptoms of atopic dermatitis. As a result, the abundance of the phylum *Firmicutes* decreased markedly in TMA-treated mice that were administered AP, while that of the phyla *Bacteroidetes* and *Proteobacteria* increased significantly (Figure 5). It was also revealed that the abundances of *Bifidobacterium spp.* and *Lactobacillus spp.*, which are involved in the onset and cure of atopic dermatitis [1,3,4,5,6], increased with the administration of AP to TMA-treated mice. In addition, the amounts of *B. fragilis*, which corrects intestinal immunity and is involved in the allergy-like eczema [32], increased with the administration of AP (Figure 6). Moreover, AP increased the amount of *C. coccoides*, which has been shown to be involved in the development of inflammatory diseases [31]. Although no reports have directly linked *C. coccoides* to the onset of atopic dermatitis, it was considered that *C. coccoides* corrects intestinal immunity, similar to *B. fragilis*. Based on the above findings, it was suggested that AP alters the gut microbiota substantially and likely prevents the onset of atopic dermatitis.

It has been reported that patients with atopic dermatitis have large amounts of intestinal *Clostridium* spp. (phylum *Firmicutes*) and small amounts of intestinal *Bifidobacterium* spp. (phylum *Actinobacteria*) [2]. However, there were no differences in the amounts of most gut microbes between the normal group and the TMA-control group (Figure 5 and Figure 6), suggesting that there is no correlation between TMA-induced atopic dermatitis-like symptoms and alteration of the gut microbiota. On the other hand, the abundance of *Bifidobacterium* spp. in the gut microbiota decreased significantly in the TMA-control group compared with the normal group. Although it has been reported that the application of hapten changes in gut microbiota [6], the reason for this is hardly known. This result suggests that skin inflammation may regulate gut microbiota and this is an important finding in considering the skin–gut axis. In the future, analysis of the effect of AP on gut microbiota in patients with atopic dermatitis is awaited.

The gut microbiota is associated with the onset of many diseases other than atopic dermatitis. For instance, it is known that in patients with obesity the abundance of the phylum *Firmicutes* is high, while that of the phylum *Bacteroidetes* is low, and the *Firmicutes*-to-*Bacteroidetes* ratio is used as an obesity index [34,35]. In addition, it was reported that the *Firmicutes*-to-*Bacteroidetes* ratio increased in diabetes [36], coronary artery disease [37], or hypertension [38]. When normal mice were fed AP, the intestinal abundance of the phylum *Firmicutes* decreased, while that of the phylum *Bacteroidetes* increased (Figure 5). These results indicate the association of changes in the gut microbiota with the antiobesity, antidiabetic, and antihypertensive action of AP.

Currently, in addition to probiotics, many prebiotics that promote the growth of intestinal bacteria are offered commercially, with the aim of preventing and treating diseases through correction of the gut microbiota. The present study—which demonstrates that polyphenols are involved in significant alteration of the gut microbiota—suggests that polyphenols may be useful as an intestinal-flora-improving food, in addition to probiotics and prebiotics. We believe that our findings provide crucial insights.

## Figures and Tables

**Table 1:** Primer sequences for real-time PCR.

Gene	Forward Primer (5’ to 3’)	Reverse Primer (5’ to 3’)
TNF-α	ATGGACACCAAACATTTCCTGC	CCAGTGGAGAGCCGATTCC
IL-6	CCCTGACAGACCCGGACTTA	GCCGAGACTGTTGTTCCATAAT
iNOS	GGCAGCCTGTGAGACCTTTG	GCATTGGAAGTGAAGCGTTTC
COX-2	CAGGGCCCTTCCTCCCGTAG	GCCTTGGGGGTCAGGGATGA
18S rRNA	GTCTGTGATGCCCTTAGATG	AGCTTATGACCCGCACTTAC

TNF-α, tumor necrosis factor-α; IL-6, interleukin-6; iNOS, inducible nitric oxide synthase; COX-2, cyclooxygenase-2.

**Table 2:** Primer sequences used for the gut microbiota.

Target	Forward Primer (5’ to 3’)	Reverse Primer (5’ to 3’)
*Firmicutes* [18]	TGAAACTYAAAGGAATTGACG	ACCATGCACCACCTGTC
*Bacteroidetes* [19]	GGGGTTCTGAGAGGAAGGT	CCGTCATCCTTCACGCTACT
*Proteobacteria* [20]	CATGACGTTACCCGCAGAAGAAG	CTCTACGAGACTCAAGCTTGC
*Actinobacteria* [21]	TGTAGCGGTGGAATGCGC	AATTAAGCCACATGCTCCGCT
*Lactobacillus* spp. [22]	TGGAAACAGRTGCTAATACCG	GTCCATTGTGGAAGATTCCC
*Bifidobacterium* spp. [23]	GGTGTTCTTCCCGATATCTACA	CTCCTGGAAACGGGTGG
*Bacteroides fragilis* [23]	ATAGCCTTTCGAAAGRAAGAT	CCAGTATCAACTGCAATTTTA
*Clostridium coccoides* [24]	GCCACATTGGGACTGAGA	GCTTCTTAGTCAGGTACCG
16S rRNA [25]	TCCTACGGGAGGCAGCAGT	GGACTACCAGGGTATCTAATCCTGTT

## Supplementary Materials

The following are available online at https://www.mdpi.com/2304-8158/9/6/773/s1, Figure S1: HPLC profile of the extract of *Acacia mearnsii* bark. Dried extract of *Acacia mearnsii* was solubilized with 60% EtOH (5.0 mg/mL). After filtration (membrane filter, 0.45 μm), 10 μL of filtrate was analyzed by analytical HPLC. Analytical HPLC was performed on a Cosmosil 5C18-ARII (Nacalai Tesque, Kyoto, Japan) column (250 × 4.6 mm, i.d.) with a gradient elution of 4−30% (39 min) and 30−75% (15 min) CH_3_CN in 50 mM H_3_PO_4_ at 35 °C (flow rate, 0.8 mL/min; detection, 230 nm; detector: Jasco photodiode array detector MD-4010, Jasco, Tokyo, Japan).
