# Supplementary material for: Acacia Polyphenol Ameliorates Atopic Dermatitis in Trimellitic Anhydride-Induced Model Mice via Changes in the Gut Microbiota
Source: Foods. 2020 Jun 11;9(6):773. doi: 10.3390/foods9060773 (PMC7353469; doi:10.3390/foods9060773)
Supplement: Supplementary file 1 [file foods-09-00773-s001.pdf]

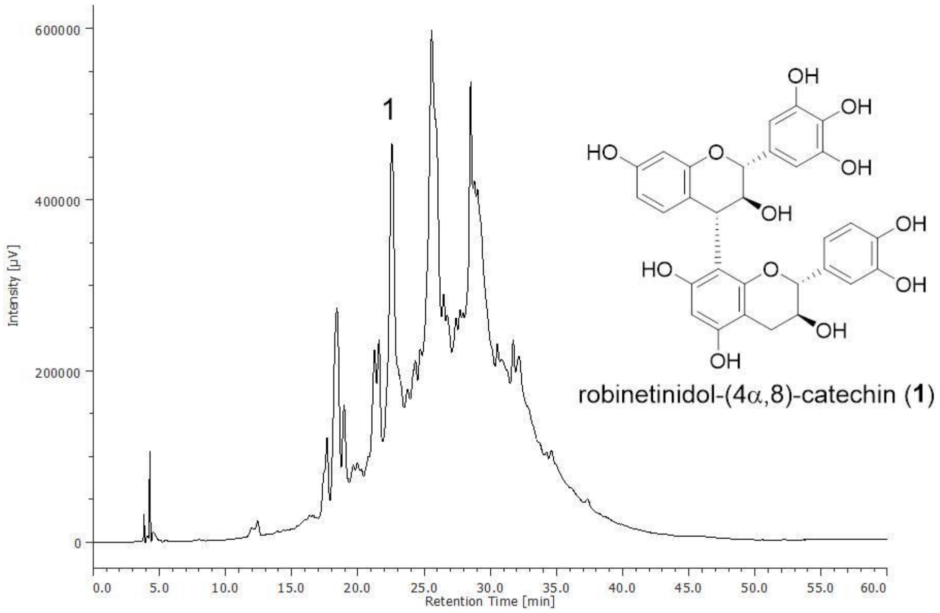

Figure S1. HPLC profile of the extract of *Acacia mearnsii* bark. Dried extract of *Acacia mearnsii* was solubilized with 60% EtOH (5.0 mg/mL). After filtration (membrane filter, 0.45 μm), 10 μL of filtrate was analyzed by analytical HPLC. Analytical HPLC was performed on a Cosmosil 5C$_{18}$-ARII (Nacalai Tesque) column (250 × 4.6 mm, i.d.) with a gradient elution of 4–30% (39 min) and 30–75% (15 min) CH_3CN in 50 mM H_3PO_4 at 35 °C (flow rate, 0.8 mL/min; detection, 230 nm; detector: Jasco photodiode array detector MD-4010, Jasco, Tokyo, Japan).
